# Clinical Impact of Low-Dose Neoadjuvant Chemotherapy in Advanced Gastric Cancer or Esophagogastric Junction Cancer: A Retrospective Analysis

**DOI:** 10.3390/life16060902

**Published:** 2026-05-27

**Authors:** Masaaki Akai, Nobuhiko Kanaya, Mikoto Shimabara, Yuta Nobunaga, Ayano Tamaki, Tsubasa Yanagihara, Toshihisa Matsumura, Kazuya Kuwada, Shoji Takagi

**Affiliations:** 1 Department of Gastroenterological Surgery, Okayama Red Cross General Hospital, 2-1-1, Aoe, Kita-ku, Okayama 700-0941, Japan; mikotoshimabara55@gmail.com (M.S.); novi1235711@gmail.com (Y.N.); ayatama.4722@gmail.com (A.T.); tsubasa.2830513@gmail.com (T.Y.); macchan10473@gmail.com (T.M.); kuwatamomoka@yahoo.co.jp (K.K.); s.takagi37@gmail.com (S.T.); 2 Department of Gastroenterological Surgery, Graduate School of Medicine, Dentistry and Pharmaceutical Sciences, Okayama University, 2-5-1, Shikata-cho, Kita-ku, Okayama 700-8558, Japan; 3 Department of Medical Technology, Graduate School of Health Sciences, Okayama University, Okayama 700-8558, Japan

**Keywords:** gastric cancer, neoadjuvant chemotherapy, low dose, S-1, oxaliplatin, SOX

## Abstract

Background. This study aimed to evaluate the efficacy and safety of low-dose neoadjuvant chemotherapy with an oral fluoropyrimidine containing tegafur, gimeracil, and oteracil (S-1) and oxaliplatin (NAC-SOX) in improving resectability and long-term outcomes in patients with advanced gastric cancer. Methods. This was a single-center, retrospective study analyzing patients with advanced gastric cancer or esophagogastric junction cancer who received NAC-SOX. (S-1: 80–120 mg/m^2^, oxaliplatin: 100 mg/m^2^) followed by gastrectomy with D2 lymphadenectomy. Clinical background, chemotherapy-related adverse effects, surgical outcomes, pathological response, and survival were assessed. Results. A total of 34 patients underwent NAC-SOX, with a median age of 74 years. The most common surgical procedure was total gastrectomy (*n* = 16). Peripheral neuropathy was the most frequent adverse effect, but no grade 4 toxicities were observed. Postoperative complications (≥CD grade 3a) occurred in 8.8% of cases, with no treatment-related deaths. R0 resection was achieved in 85.3% of cases, and the pathological complete response rate was 20.6%. The 3-year recurrence-free and overall survival rates were 71.3% and 83.2%, respectively. Conclusions. Low-dose NAC-SOX demonstrated favorable efficacy and safety, achieving high R0 resection and pathological response rates. Further prospective studies are needed to optimize treatment strategies.

## 1. Introduction

Gastric cancer remains a leading cause of cancer-related mortality worldwide, necessitating the continuous development of more effective and personalized therapeutic strategies [1,2,3]. Recent advancements in molecular biology have provided deeper insights into the tumor microenvironment and the heterogeneity of gastric cancer, offering a new biological context for optimizing treatment paradigms [4]. In this landscape, neoadjuvant chemotherapy (NAC) has emerged as a crucial component of perioperative management, particularly for locally advanced gastric cancer or esophagogastric junction cancer, aiming to improve resectability and long-term survival outcomes.

Perioperative chemotherapy has been widely recognized as an effective treatment approach for various cancers [5,6,7,8]. In the case of gastric cancer, postoperative adjuvant chemotherapy is currently the standard treatment in Japan, aiming to reduce recurrence risk and improve overall survival [3]. S-1 is a fixed-dose combination agent comprising three pharmacologically distinct compounds: tegafur, a prodrug of 5-fluorouracil (5-FU) that is converted into its active form in the body; gimeracil, which inhibits dihydropyrimidine dehydrogenase (DPD) to prevent the rapid metabolic degradation of 5-FU, thereby maintaining high plasma concentrations; and oteracil, which is a poorly absorbed inhibitor of orotate phosphoribosyltransferase that localizes within the gastrointestinal tract to minimize 5-FU-related toxicities. However, neoadjuvant chemotherapy (NAC) with S-1/cisplatin is only recommended for patients with bulky N or para-aortic lymph node metastases [3]. The rationale for NAC is that it can reduce tumor burden before surgery, potentially increasing the likelihood of complete resection while also addressing micrometastases at an early stage. To further explore the role of NAC in gastric cancer, the JCOG1509 clinical trial is currently evaluating the efficacy and safety of S-1 and oxaliplatin (SOX) as a neoadjuvant regimen for patients with clinical stage IIB–III disease [9]. While the standard dose of oxaliplatin is 130 mg/m^2^ in Japan, many patients in real-world clinical practice are elderly or have comorbidities, making the management of treatment-related toxicities a significant challenge. Therefore, evaluating the clinical significance of a reduced-dose regimen (100 mg/m^2^) is crucial, as it may provide a more tolerable yet effective alternative for this vulnerable population without compromising surgical outcomes.

At our institution, we have adopted a treatment strategy involving low-dose NAC-SOX following sufficient induction chemotherapy for patients with advanced gastric cancer and esophagogastric junction cancer. This approach is based on the premise that NAC may help control disease progression, improve resectability, and ultimately enhance long-term survival outcomes. In this study, we analyze and report the clinical outcomes of patients who received NAC-SOX, with a focus on its efficacy, safety, and impact on overall prognosis. By sharing these findings, we aim to contribute to the growing body of evidence supporting the use of NAC in the management of advanced gastric cancer.

## 2. Materials and Methods

### 2.1. Samples and Data Collection

This study was a single-arm, non-comparative, single-center, retrospective clinical study. This study analyzed patients with gastric cancer with large Type 3 or 4 tumors, T4 or upper-body T3 lesions, and nodal metastasis, with an ECOG performance status (PS) of 0 or 1. Following informed consent, NAC-SOX (oxaliplatin 100 mg/m^2^ on day 1, oral S-1 80–120 mg/m^2^ twice daily for two weeks every three weeks) was performed. S-1, an oral fluoropyrimidine derivative, is a fixed-dose combination of tegafur, gimeracil, and oteracil potassium in a molar ratio of 1:0.4:1. Tegafur is a prodrug of fluorouracil, 5-FU. Gimeracil inhibits the degradation of fluorouracil by inhibiting dihydropyrimidine dehydrogenase. Oteracil is a poorly absorbed drug, staying largely within the gut where it inhibits orotate phosphoribosyltransferase thereby lowering evolution of 5-FU levels in the gut. The number of treatment cycles was mainly three, though the dose was paused due to patients’ health conditions. Subsequently, laparoscopic distal or total gastrectomy with D2 lymphadenectomy was performed 4–8 weeks after the last NAC-SOX cycle. Clinical background, NAC-SOX-related adverse effects, perioperative outcomes, and long-term postoperative outcomes were evaluated. In this study, we defined good response as a pathological tumor regression grade of 2b or 3, representing a significant histological response to neoadjuvant chemotherapy. This study was conducted in accordance with the principles of the Declaration of Helsinki, and the protocol was approved by the Institutional Review Board of Okayama Red Cross Hospital (No. 2022-37). Patients provided written informed consent for publication of their details.

### 2.2. Statistical Analysis

Data are presented as totals, medians (range), or percentages. Statistical analyses were performed using EZR (Saitama Medical Center, Jichi Medical University, Saitama, Japan), which is a graphical user interface for R (The R Foundation for Statistical Computing, Vienna, Austria). Survival analysis was conducted using the Kaplan–Meier method. Univariate analysis was conducted using logistic regression analysis. 

## 3. Results

### 3.1. Clinical Background of Patients with Neoadjuvant Chemotherapy

A total of 34 patients received NAC-SOX. The characteristics of these patients are summarized in Table 1. The median age was 74 years (range from 39 to 81 years). Among the patients, 29 were men, and 5 were women. Of the 10 patients with esophagogastric junction (EGJ) cancer, 2 were classified as clinical stage T2. Then, eleven of 34 patients had clinical stages I or II. Four cases of multiple organ invasion were noted, including two involving the transverse colon and two involving the pancreas. The most common surgical procedure was total gastrectomy (*n* = 16), followed by subtotal esophagectomy (*n* = 1) and lower esophagectomy/proximal gastrectomy (*n* = 4).

### 3.2. Adverse Effects of Neoadjuvant Chemotherapy in Advanced Gastric Cancer

The adverse effects of NAC are shown in Table 2. Peripheral neuropathy was the most frequently observed side effect, although no serious complications were recorded. Thrombocytopenia was observed in 23.5% of the cases, with 11.8% experiencing complications requiring chemotherapeutic dose reduction or delayed surgery. Nevertheless, curative resection was feasible for all patients.

### 3.3. Surgical Outcomes After NAC-SOX in Advanced Gastric Cancer

The short-term outcomes of surgical treatment are presented in Table 3. The median intraoperative blood loss volume was 150 mL (0–1100 mL). A total of 85.3% of patients underwent R0 surgery, and positive ascites cytology accounted for the R1 cases. Postoperative complications were graded according to the Clavien–Dindo classification system, a standardized method for classifying surgical complications by the therapeutic interventions required to treat them. Postoperative complications (Clavien–Dindo class 3a or higher) occurred in three patients (8.8%), including one case of anaphylactic shock after tube removal, one case of anastomosis leakage, and one case of postoperative bleeding. The median length of postoperative hospital stay was 12.5 days (8–52 days).

### 3.4. Pathological Outcomes of NAC-SOX in Advanced Gastric Cancer

Postoperative pathology results are summarized in Table 4. Seven patients (20.6%) achieved pathological complete response (CR). Twenty-four (70.6%) pathological responders at grade 1b or higher responded to chemotherapy. Univariate analysis showed that the factors associated with good response (grade 2b–3) to NAC-SOX were tumor location in the upper stomach and macro type 2, albeit with no statistical. The pretreatment T factors in the CR cases were T4 in six cases and T3 in one case.

### 3.5. Long-Term Outcomes of NAC-SOX in Advanced Gastric Cancer

**Figure 1:**
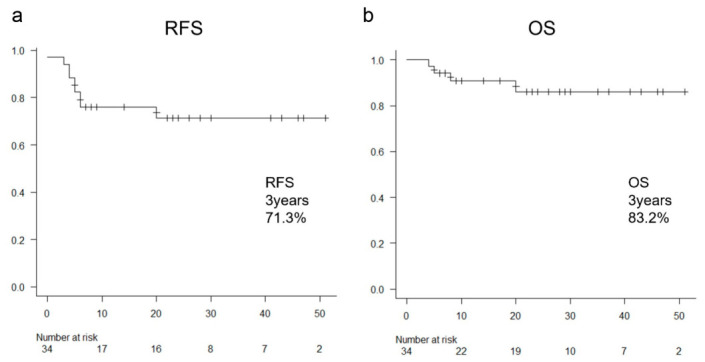
Long-term outcomes of NAC-SOX. (**a**) Kaplan–Meier showing recurrence-free survival (RFS) rate at 3 years after surgery and (**b**) overall survival (OS) rate.

Long-term outcomes after curative (Median follow-up: 21 months) are shown in Figure 1. The recurrence-free survival rate at 3 years after surgery was 71.3%, whereas the overall survival rate was 83.2% (Figure 1). 

## 4. Discussion

This study demonstrated the efficacy of NAC-SOX for locally advanced gastric cancer and esophagogastric junction cancer. Compared with previous reports, the response rate and the clinical safety were more favorable. Additionally, the short- and long-term outcomes after NAC-SOX were acceptable.

Recently, the clinical benefits of NAC in patients with gastric cancer were discussed in the world based on the clinical trials with other cancers. A phase 3 trial with NAC for locally advanced gastric cancer previously revealed negative outcomes in the JCOG0501 study [10,11]. Then, Japanese guidelines limit the indications for NAC for locally advanced gastric cancer, which is in line with our study findings [3]. However, recent reports on the efficacy of NAC both in Japan and overseas have led to a reevaluation of the value of NAC [12,13,14,15,16]. In Europe and the United States, NAC is the standard treatment for advanced gastric cancer. The efficacy of preoperative 5-fluorouracil-leucovorin-oxaliplatin-docetaxel (FLOT) therapy was reported as a CR rate of 16% according to the FLOT trial [12,13]. In Korea, the efficacy of preoperative docetaxel-oxaliplatin-S-1 has been reported, with a CR rate of 14.6% [14]. The efficacy of SOX has also been reported; the trials that compared NAC-SOX and NAC-FLOT therapies showed a CR or subtotal response rate of 32.4% vs. 20%, respectively [15]. Furthermore, in Japan, the KSCC1601 trial reported the safety and efficacy of NAC-SOX, with a CR rate of 9.5% [17]. Ota et al. also reported that the CR rate of NAC-SOX was 16.7% [16]. The JCOG1509 trial, a large-scale multicenter clinical trial investigating the efficacy of NAC-SOX, is currently ongoing. The results of this trial are awaited in the future.

The perioperative SOX regimen is increasingly recognized as a standard of care for locally advanced gastric cancer, with the randomized trial by Yu et al. demonstrating its non-inferiority to FOLFOX [18]. Furthermore, recent evidence by Kawabata et al. emphasizes the feasibility and safety of S-1-based neoadjuvant chemotherapy in older patients [19]. The importance of this study lies in the chemotherapeutic dose intensity of oxaliplatin. In this study, the median age of patients was 74 years, reflecting a real-world population that often struggles to tolerate standard-dose intensity. Our findings suggest that a 100 mg/m^2^ dose of oxaliplatin is particularly significant in an aging society. Compared to previous trials like KSCC1601, which utilized the standard dose, our reduced-dose strategy achieved a reasonable CR rate with an improved safety profile. Furthermore, while standard-dose regimens such as FLOT have reported complete response rates of 16%, our results suggest that high doses do not always yield superior clinical responses. In the future, it will be necessary to identify patients who would benefit from higher doses.

However, there are still challenging issues related to NAC for locally advanced gastric cancer. According to the European Society of Medical Oncology guidelines, NAC is indicated for all advanced gastric cancers [20]. Differing from this, the Japanese guidelines emphasize postoperative adjuvant chemotherapy for stage III gastric cancer [3]. NAC might be particularly beneficial for patients undergoing total gastrectomy, as postoperative doublet chemotherapy is often challenging due to poor physical recovery and nutritional intake. Furthermore, good responders to NAC could prevent total gastrectomy and allow for R0 resection. Therefore, we recommend NAC-SOX in patients with advanced gastric cancer in the upper stomach. Our results suggest that NAC-SOX is more effective against cancer in the upper gastric body.

This study had some limitations. First, this was a single-center retrospective study. Second, the study had a small sample size; hence, the outcomes of this advanced therapy should be prospectively investigated in future clinical trials. Third, the indications of NAC in this study differed from those used in other large-scale clinical trials, making the results unsuitable for direct comparison. Finally, this study included tumors that were not as advanced in stage as those in other trials, which might have resulted in better treatment response determination. Nevertheless, the benefits of NAC are significant, and it remains an effective treatment. Further studies are required to clarify the indications of NAC.

## 5. Conclusions

The clinical significance of this study demonstrates that a reduced-dose NAC-SOX regimen can be a viable treatment strategy for advanced gastric cancer, especially in elderly patients.

## Figures and Tables

**Table 1:** Characteristics of the patients. EGJ, esophagogastric junction; U, upper; M, middle; L, lower; NAC-SOX, neoadjuvant chemotherapy of S-1 and oxaliplatin; DG, distal gastrectomy; TG, total gastrectomy; PG, proximal gastrectomy; subE, subtotal esophagectomy; LE + PG; lower esophagectomy and proximal gastorectomy.

Characteristics	*n* = 34
Sex Male/Female	29/5
Age, median (range)	74 (39–81)
Location	
EGJ/U/M/L	10/7/9/8
Clinical diagnosis	
T2/T3/T4a/T4b	2/3/22/4
N0/N1/N2/N3a-	14/8/5/7
cStage 1/2/3/4a/4b	2/9/15/4/4
Number of NAC-SOX cycles 2/3/4	6/25/3
Operation DG/TG/PG/subE/LE + PG	12/16/1/1/4

**Table 2:** Adverse events of NAC-SOX.

	Any Grade *n* = 34 (%)	Grade 3–4 *n* = 34 (%)
Leucopenia	4 (11.8%)	0 (0%)
Neutropenia	9 (26.5%)	0 (0%)
Febrile Neutropenia	-	0 (0%)
Anemia	14 (41.2%)	0 (0%)
Thrombocytopenia	8 (23.5%)	4 (11.8%)
Anorexia	14 (41.2%)	0 (0%)
Nausea	11 (32.4%)	0 (0%)
Vomiting	2 (5.9%)	0 (0%)
Diarrhea	3 (8.8%)	0 (0%)
Peripheral neuropathy	21 (61.8%)	0 (0%)

**Table 3:** Surgical outcomes after NAC-SOX. CD, Clavien–Dindo.

	*n* = 34
Operation time, median (range)	331 (295–579)
Blood loss, median (range)	150 (0–1100)
Resection R0/R1/R2	29 (85.3%)/3/2
Complication	
Any CD grade	12 (35.3%)
≥CD grade 2	9 (26.5%)
≥CD grade 3a	3 (8.8%)
Anastomosis leakage	1 (2.9%)
Postoperative hospital stay, median (range)	12.5 (8–52)

**Table 4:** Postoperative pathological outcome after NAC-SOX. tub, tubular adenocarci noma; poor, poorly differentiated adenocarcinoma; sig, signet-ring cell carcinoma; muc, mucinous adenocarcinoma; CR, complete response.

	*n* = 34
Pathological diagnosis	
T0/T1-2/T3/T4a/T4b	7/7/10/8/2
N0/N1/N2/N3	20/3/3/8
pStage 0/1/2/3/4	7/7/8/6/6
Histological type	
tub1/tub2/por/sig/muc	5/12/9/1/1
Grade1a/1b/2a/2b/3	10/10/3/4/7
Pathological responder(grade1b-3)	24 (70.6%)
Pathological CR (grade3)	7 (20.6%)

## Data Availability

All data generated and/or analyzed during our study are available from the corresponding author upon reasonable request.

## Abbreviations

The following abbreviations are used in this manuscript:

NAC	Neoadjuvant chemotherapy
SOX	S-1 and oxaliplatin
PS	Performance status
EGJ	Esophagogastric junction
CR	Complete response
